# Epidemiology of Autism Spectrum Disorders: A Review of Worldwide Prevalence Estimates Since 2014

**DOI:** 10.3390/brainsci10050274

**Published:** 2020-05-01

**Authors:** Flavia Chiarotti, Aldina Venerosi

**Affiliations:** Reference Center for the Behavioural Sciences and Mental Health, Italian National Institute of Health, 00161 Rome, Italy

**Keywords:** prevalence estimate, autism, predictors, surveillance review

## Abstract

The prevalence of Autism Spectrum Disorder (ASD) has increased dramatically in recent decades, supporting the claim of an autism epidemic. Systematic monitoring of ASD allows estimating prevalence and identifying potential sources of variation over time and geographical areas. At present, ASD prevalence estimates are available worldwide, coming either from surveillance systems using existing health and educational databases or from population studies specifically performed. In the present article, we present a review of the ASD prevalence estimates published since 2014. Data confirm a high variability in prevalence across the world, likely due to methodological differences in case detection, and the consistent increase of prevalence estimates within each geographical area.

## 1. Introduction

In the last decades, a large increase in the prevalence of Autism Spectrum Disorder (ASD) has been observed, generating claims about an “epidemic” of autism [1,2]. Correct estimates of ASD prevalence rates are important, firstlyin order to determine the economic and health services burden of this condition and to allocate sufficient funding and adequate services for children and adults with ASD and their families. A growing population of people with ASD implies the necessity of increased service availability including training of professionals, as well as identification of additional resources that can emerge by the recognition of cases in the population [3]. Furthermore, accurately determining ASD prevalence can help to understand which groups are exposed to disparities in healthcare access for developmental evaluations [4], besides being more at risk for ASD due to geographical and environmental factors [5].

Studies that estimate ASD prevalence result in wide variability of prevalence rates that call for paying attention on possible reasons for the observed changes in prevalence, and advice for caution when claiming that there is an autism epidemic [2,6].

One important source of variation in prevalence estimates are the methodological differences in case definition and case-finding procedures. In particular, some studies are carried out on existing administrative databases such as special education data, health or social records of national registers for case identification, or specific condition registers (defined as “administrative data” when relying on one database, or “multisource” when combining data from multiple databases). Other studies rely on a two-stage or multistage approach to identify cases in underlying populations; the first stage is often based on questionnaire requesting behavioural descriptions or checklist based on DSM, where informants could be in turn teachers, parents or health professionals (defined as “ad hoc studies”). Finally, some studies are surveys based on interviews to parents or teachers, who are required to state if the child presents a condition that can be related to ASD (defined as “reports”). Obviously, sample size and catching area represent further characteristics of the studies that can affect prevalence estimates [7]. Indeed, surveyed areas vary in terms of service development as a function of the specific educational or health care systems of each country and of the year of the study [6]. Moreover, socio economic factors [8,9] and autism awareness [10] can influence assessment of the case and consequently prevalence estimate.

Case definition is the other challenge that affects prevalence estimate. Diagnostic category (AD, ASD, PDD), as well as age range considered are very important sources of prevalence estimate variation [7]. Changing definitions and labelling practices that change over time, as in the case of the introduction of diagnostic manuals’ revisions, can produce change in labelling but also “diagnostic substitution” whereby similar symptoms can be classified under different disabilities during different time periods [11,12]. Lastly, cultural influence can affect the definition of case causing differences in the estimation of prevalence in different ethnic/cultural groups [13,14].

In the present paper, we present a brief narrative review of the most recent ASD prevalence estimates worldwide. We describe evidences according to two main criteria, i.e., the geographical setting and the case-finding procedure of the study. Finally, we attempt to demonstrate if these criteria act as predictive factors for underestimating or overestimating prevalence figures.

## 2. Prevalence Estimates

Many prevalence studies have been performed worldwide since 1966. In 2012, Elsabbagh et al. [7] published a comprehensive review of the studies performed until 2012: these studies differed with respect to diagnostic category, diagnostic criteria, age at prevalence evaluation, extent of the targeted geographical area, and source of data on the diagnoses. These methodological differences, together with the large time span (almost 50 years from the first to the last study included in the review), at least partly account for the large differences observed in the estimated prevalence. Overall, estimates ranged from 0.19/1000 to 11.6/1000. The former estimate refers to the Autistic Disorder (AD), diagnosis based on Rutter’s criteria (1978), age range 0–14 years, geographical area of West Berlin (Germany), and on data extracted from the registry of the university clinic of child psychiatry and/or the German Society for Autistic Children (1986). The latter refers to the Pervasive Developmental Disorder (PDD), diagnosis based on ICD-10, age range 9–10 years, geographical area of South Thames (UK), ad hoc study (2006). Taking into account the diagnostic category, the median prevalence estimates were 1.00/1000 for AD (from 0.19/1000 in Germany to 7.26/1000 in Sweden) and 6.16/1000 for PDD (from 3.00/1000 in Denmark to 11.6/1000 in UK). When considering PDD, the median prevalence was similar to the USA overall ASD prevalence estimated in 2000–2002, but much lower than the USA prevalence estimates since 2006.

In 2014, Tsai updated the review by [7], but only negligible differences emerged in the median prevalence estimates, which were confirmed to be 1.32/1000 for AD (from 0.19/1000 in Germany to 7.26/1000 in Sweden) and 6.19/1000 for PDD/ASD (from 3.00 in Denmark 2002 to 12.3/1000 in Netherlands) [15]. The review by Tsai included almost all papers evaluated by [7], specifically 59/59 = 100% for AD and 33/35 = 94.3% for PDD prevalence studies. In his review, Tsai examined 15 and 28 additional studies estimating AD and PDD/ASD prevalence, respectively. Table 1 reports a summary of the results of the reviews by [7,15].

Since the publication of the reviews by Elsabbagh et al. and Tsai, more prevalence studies have been performed worldwide. In the following, we report the prevalence studies published since 2014 according to the geographical area of reference. Some studies yield ASD prevalence estimate at different calendar year and/or in different age classes: where possible, we selected the more recent estimate of prevalence that referred to age 8. The list of studies with details and prevalence estimates are presented in Table 2, Table 3 and Table 4.

### 2.1. Europe

In Sweden, a prevalence study was performed in 2011 based on data from the Stockholm Youth Cohort (SYC) [16]. SYC is a record-linkage study collecting data longitudinally from 2001 to 2011 on all children from 0 to 17 years of age residing in Stockholm County in any time in the specified period; a multisource case ascertainment methodology was used to assess the presence of an ASD diagnosis. An overall large increase of ASD prevalence was observed between 2001 and 2011. Specifically, in children aged 0–17 years, the prevalence moved from 4.20/1000 in 2001 to 14.4/1000 in 2011, with an increase of almost 250%. This increase was mainly due to the huge increase of the ASD prevalence observed in children/adolescents without intellectual disability (almost +700%, from 1.40/1000 in 2001 to 11.0/1000 in 2011), while the increase of ASD prevalence in children/adolescents with intellectual disability was much lower (about +20%, from 2.80/1000 in 2001 to 3.40/1000 in 2011).

In Poland (West Pomeranian—WP—and Pomeranian—P—regions), Skonieczna-Zydecka and collaborators [17] estimated ASD prevalence in 2010–2014 on children from 0 to 16 years of age based on data obtained from both government and private institutions concerning ASD diagnoses and certificates of disability. The prevalence estimates in children of all ages were similar in the two regions (3.24 vs. 3.76/1000 in WP and P, respectively). In both regions, the highest prevalence was observed in children from 4 to 7 years of age (5.35 and 5.25/1000 in WP and P, respectively), yielding an overall estimate of 5.29/1000 in this age class.

In Germany, Bachmann et al. [18] conducted a study aimed at estimating at a national level the administrative prevalence of ASD in individuals aged up to 24 years, using inpatient and outpatient claims data of National health insurance from 2006 to 2012. The 2012 estimates were used to detect differences in prevalence among age groups. Children from 6 to 11 years of age showed the highest prevalence, estimated at 6.00/1000.

In the European Union, 14 countries have participated to the European project “Autism Spectrum Disorders in Europe (ASDEU)”: Spain (programme lead), Austria, Belgium, Bulgaria, Denmark, Finland, France, Iceland, Ireland, Italy, Poland, Portugal, Romania, and United Kingdom. Among the goals of the project, there was the estimation of the prevalence of ASD in children aged 7–9 years in 2015. Four countries estimated the prevalence of ASD in 8 years old children using nationwide registry data (Denmark, Finland, and Iceland) or regional statistics (France); prevalence estimates were very different among countries, ranging from 4.76/1000 in South-Eastern France to 31.3/1000 in Iceland (for details, see Table 2) [19].

Eight countries (Austria, Bulgaria, Ireland, Italy, Poland, Portugal, Romania, and Spain) performed ad hoc studies following a shared protocol that required the participation of schools, teachers and parents. Teachers and parents were required to fill in questionnaires (Teacher’s nomination and Social Communication Questionnaire, respectively) in order to screen children at risk of having ASD. The children at risk successively underwent a clinical assessment to confirm (or not) the diagnosis of ASD. Until now, only the results of the ASDEU ad hoc study performed in Italy have been published [20]. This study yielded a prevalence estimate of 7.99/1000 when using just the number of children certified with ASD or with other neurodevelopmental disorders in comorbidity with ASD. This prevalence rose to 10.4/1000 when including children identified through the screening procedure, and to 11.5/1000 based on a probabilistic calculation to adjust for non-responses. This estimate was much higher than those based on regional administrative databases storing data on services provided by Child and Adolescent Mental Health Units in Italy, namely SMAIL in Piemonte and ELEA in Emilia Romagna regions. These regional databases yielded in 2016 prevalence estimates of 4.20 and 4.30/1000 in children aged 6–10 years and 6.20/1000 and 5.50/1000 in children aged 3–5 years (Piemonte and Emilia-Romagna regions, respectively). A more recent regional estimate in 2018 based on administrative data from Abruzzo region yielded a higher prevalence estimate of 7.98/1000 in the age class 6–8 years, and quite similar prevalence estimate of 5.74/1000 in the age class 3–5 years [21]. These data suggest that prevalence estimates based on data extracted from registries built to meet administrative informative needs are on average lower than estimates coming from ad hoc studies, mainly when a two-phase ascertainment design (screening and diagnosis confirmation) is used. UK data support this insight. The high prevalence observed in 2006 in South Thames, consistent with that estimated in Cambridgeshire in 2003–2004 by a school-based population study (15.7/1000) [43], was very different from the prevalence in children aged 8 years estimated by administrative data from the UK General Practice Research Database [13]. The database, activated in 1990 and storing medical records from the general practitioners, produced a much lower prevalence (from 3.58/1000 in 2004 to 4.09 and 3.90/1000 in 2009 and 2010, respectively), even lower than the median prevalence estimate reported by [7].

In Spain, a two-phase cross-sectional study in the framework of EPINED project was performed in Tarragona (year of study performance not specified), yielding prevalence estimates of 15.5/1000 and 10.0/1000 in the age classes 3–5 years and 10–12 years, respectively [22]. At about the same time, a study was performed using data from the Catalan Public Health Service on children aged 2 to 17 years. The estimated ASD prevalence in 6–10 years old children for 2017 was 11.8/1000, a rather high value for an estimate based on administrative data [23].

### 2.2. Middle-East

Few studies have been performed up to now in Middle East countries, generally yielding prevalence estimates lower than Western Countries.

In Iran, the most updated estimate of ASD prevalence comes from a study that is part of a large-sample national population based epidemiological study concerning psychiatric disorders among Iranian children and adolescents aged 6–18 years [24]. The weighted ASD prevalence estimate for 6–18 years old subjects (computed from data reported in the paper) is approximately 1.60/1000, lower than less recent estimates from United Arab Emirates (2.90/1000 for 0–14 year children) [44], and Israel (4.80/1000 for 1–12 year children) [45].

As already reported in the review by [7], in 2010 a very low countrywide prevalence of 0.14/1000 had been estimated in children aged 0–14 years in the Sultanate of Oman [46]. This prevalence possibly reflected a low capacity of detecting children with ASD more than an actual low proportion of children affected. The lack of biological markers of ASD and the low availability of health services for the diagnosis of and the intervention on children with ASD were examined as factors that may account for the low prevalence [47]. More recently, Al-Mamri et al. performed a multicentre study aimed at updating the estimate of ASD prevalence among Omani children, using data retrieved from the three main centres for the diagnosis of ASD in the Sultanate of Oman in the period December 2011–December 2018 [25]. The new estimate was 2.04/1000 in the overall group of children (0–14 years of age); even if it is almost 15-fold higher than the previous one, it is still very low with respect to most of the estimates worldwide. Within the country, the highest prevalence was observed in Muscat (3.65/1000) with a prevalence in boys 3.4-fold higher than in girls (3.12/1000 vs. 0.91/1000, respectively).

Qatar is a country with a small population (2.7 million) characterized by a very high literacy rate, free and mandatory school attendance, and free healthcare for nationals and residents. A cross-sectional two-phase survey was conducted from 2015 to 2018 to estimate ASD prevalence in children aged 6 to 11 years [26]. The total prevalence (deriving from prevalence of already- and newly-diagnosed cases) was estimated at 11.4/1000, a value much higher than those observed in the other middle-east countries.

In Lebanon, a cross-sectional study was performed in 2014 in nurseries of Beirut and Mount Lebanon, to estimate ASD prevalence in toddlers aged 16–48 months using M-CHAT and a short structured questionnaire developed in the study [27]. Since it was not possible to conduct a follow-up interview to ascertain the M-CHAT results, the proportion of toddlers with a positive result at the M-CHAT was calculated, and corrected by an estimated positive predictive value, yielding a final ASD prevalence of 15.3/1000. This value is quite high and similar to the prevalence estimated in western countries.

### 2.3. Asia

Qiu et al. [48] have published a systematic review and meta-analysis of studies on prevalence of ASD in South Asia (Sri Lanka, 2009; Bangladesh, 2009 and 2018; India, 2017; Nepal, 2018), East Asia (South Korea, 2011; China, 2011 and 2014), and West Asia (corresponding to the Middle East region: Iran, 2012; Israeli, 2013; Lebanon, 2016). Prevalence estimates show a very large variability across countries, ranging from very low values estimated in Iran, 2012 (0.63/1000) and Bangladesh, 2018 (0.76/1000), to low values estimated in India, 2017 (1.53/1000), China, 2011 (1.77/1000), India, 2017 (2.19/1000), China, 2014 (2.75/1000), Nepal, 2018 (3.42/1000), Israeli, 2013 (4.80/1000). On the contrary, large values were estimated in Bangladesh, 2009 (8.42/1000) and Sri Lanka, 2009 (10.7/1000), and very large values in Lebanon, 2016 (15.3/1000) and South Korea, 2011 (26.4/1000).

The Qiu’s review did not include some recent studies performed in China [29,30,31], in Japan [32], and in India [33]. In China, Yang et al. (2015) [31] performed ASD assessment in 2014 in toddlers (3.8 to 4.8 years of age) who attended mainstream kindergarten in Shenzhen, estimating ASD prevalence at 26.2/1000. Sun et al. (2015) [29] evaluated ASD prevalence in children aged 6 to 11 years from two mainstream schools in Beijing, yielding an estimate of 11.9/1000. In 2019, Sun et al. [30] estimated ASD prevalence in three cities (Jilin, Shenzhen, and Jiamusi) at December 2013, using data from mainstream school only in all the cities and from the whole population in Jilin. Estimates based on mainstream school population were much lower than prevalence estimated in Beijing, ranging from 1.46/1000 in Jilin to 4.23/1000 in Shenzhen, the latter much lower than that estimated from toddlers. On the contrary, the estimate based on the overall population (from mainstream and special schools, private intervention centres, and community not attending school) in Jilin was 10.8/1000, nearer to the estimates from Beijing and from Western countries.

In Japan, a community sample survey was performed to estimate prevalence of neurodevelopmental disorders (NDD) and their co-occurrence in children aged 6–9 years, using questionnaires administered to parents and teachers [32]. The estimated prevalence of ASD, alone or in co-occurrence with other NDD, was 19.0/1000 based on parent’s reports, and rose to 93.0/1000 based on teacher’s reports. The latter was quite large, much larger than what observed in all other countries. In addition, the agreement rate between parent and teacher estimates was very low, suggesting that teacher’s estimate could be largely overestimated and unreliable.

With regard to South Asia, Poovathinal et al. [33] performed a community-based survey in 2011–2012 in Kerala, South India. The study was part of a two-phase epidemiologic survey on chronic diseases performed on the entire regional population. The ASD prevalence in children from 6 to 10 years of age (i.e., the age class showing the highest prevalence) was estimated at 5.05/1000.

Finally, Hoang et al. [37] conducted a two-phase cross-sectional study (screening with M-CHAT and confirmation by clinical assessment) in toddlers from 18 to 30 months of age in Vietnam (Hanoi and Northern provinces). The estimated prevalence was 7.52/1000, obtained as proportion of children confirmed to have ASD on the number of children who underwent ASD assessment. However, the percentage of children undergoing ASD assessment after screening by M-CHAT was 100% in screen-positive children and 2% only in screen-negative ones. In addition, the percentage of ASD confirmation was 52.2% and 0.3% in screen-positive and screen-negative children, respectively. When taking into account the difference in the rate of ASD assessment following M-CHAT screening, and the difference in the rate of ASD confirmation between the two groups of children, the prevalence estimate rose to 10.8/1000, a value much higher than the values from previous studies in the same country, and much more similar to estimates in the Western countries.

### 2.4. Australia & New Zealand

In Australia, Randall et al. published in 2016 a study performed within the Longitudinal Study of Australian Children (LSAC) framework [38]. Data on the parent-reported ASD diagnoses were collected for children belonging to two different cohorts, recruited in 2004 at birth (B-cohort, years of age 2004) and in kindergarten (K-cohort, years of birth 1999–2000). Data were obtained from two different waves of the LSAC, referring to children aged 6–7 years in 2010–2011 (for B-cohort, wave 4) and in 2005–06 (for K-cohort, wave 2). Estimated ASD prevalence in 2005–2006 was 14.1/1000, and rose to 25.2/1000 in 2010–2011. Both prevalence estimates were much higher than the previous estimate of 3.92/1000 found by Icasiano et al. [49] in children aged 2–17 years during 2002, living in the Barwon region in Australia. It has to be noted that the estimate by Icasiano et al. refers to children and adolescents in a wider range of age, thus including diagnoses performed in different calendar years that are also affected by different capability of recognizing ASD. Secondly, researchers did not perform ad hoc case ascertainment, basing the estimate of prevalence on formal diagnoses of ASD made prior to data collection. As already reported for the use of data extracted from registries in UK, Italy, and China, prevalence estimates based on existing data are usually lower than estimates coming from ad hoc studies with active ascertainment of cases, and the gap is even larger with respect to estimates based on (often uncontrolled) parent-reported diagnoses (see USA below).

### 2.5. North America

ASD prevalence estimates have been produced in three regions of Canada (Newfoundland and Labrador, NL; Prince Edward Island, PEI; Southeastern Ontario, SO) from 2003 to 2010, using data from the National Epidemiologic Database for the Study of Autism of Canada (NEDSAC) [39]. A general increase of prevalence across years was observed in the three regions, with large differences in prevalence among regions. In children aged 6–9 years, in NL region the prevalence increased from 5.20/1000 in 2003 to 10.8/1000 in 2008. In the PEI region, the prevalence passed from 5.88/1000 in 2003 to 6.13/1000 in 2008, and 9.99/1000 in 2010, and in SO region, from 8.34/1000 in 2003 to 12.4/1000 in 2008 and 16.2/1000 in 2010. Prevalence has been also estimated from2000 to 2015 using data from Quebec Integrated Chronic Disease Surveillance System (QICDSS) [40]. All residents in Quebec for at least one day from January 1, 1996, to March 31, 2015, and aged up to 24 years, were considered eligible for the prevalence study. Physician claims or hospital discharges from 2000 to 2015 reporting a diagnosis of ASD, Rett syndrome or childhood disintegrative disorder at ICD-9 or ICD-10, were used to classify the patient as having ASD. The lifetime prevalence for children aged 1 to 17 years was estimated at 1.50/1000 in 2000–2001, rising up to 12.2/1000 in 2014–2015. In general, a large variability in prevalence rates was observed among sub-areas, with higher prevalence in Montreal metropolitan area and lower in semi-urban and smaller regions.

In the USA, the Centers for the Disease Control and Prevention (CDC) launched in 2000 the Autism and Developmental Disabilities Monitoring (ADDM) Network, with the aim of tracking the number and characteristics of children with ASD in multiple communities in the United States. The ADDM Network is a multisite, multiple-source, record-based surveillance system, providing the most updated and comprehensive estimates of prevalence of ASD and other developmental disabilities in children aged 8 years; this age was chosen because of the peak ASD prevalence observed among elementary-school-aged children. Since 2010, the prevalence is estimated also in children aged 4 years. Prevalence estimates are given from 2000 and every two years (except for 2004); the most recent estimates refer to 2016 [12,41,50,51,52,53,54,55]. The ADDM Network program uses the systematic screening of databases/registries (related to health, service provision for developmental disabilities, special education) in order to extract information concerning behaviours possibly associated to developmental disorders, building a multi-information record for any child of the specific age class living in the reference geographical area. Information collected in the child’s record is then examined to evaluate if the child can be diagnosed with ASD or other developmental disability, based on the Diagnostic and Statistical Manual of Mental Disorders, Fourth Edition, Text Revision (DSM-IV-TR). In 2014, 81% of the overall population underwent diagnostic evaluation by both DSM-IV-TR and DSM, Fifth Edition (DSM-5).

Table 5 and Table 6 show the USA prevalence estimates in the overall examined populations of children aged 8 and 4 years, respectively, and in subgroups of children based on sex and Ethnicity.

As can be seen, in children aged 8 years the prevalence raised steadily from 6.60/1000 in 2002 to 14.7/1000 in 2010, remained constant from 2010 to 2012, and then raised again, arriving at 16.8/1000 in 2014 and 18.5/1000 in 2016, with an increase of 181% with respect to 2002. ASD prevalence also increased in children aged 4 years, passing from 13.4/1000 in 2010 to 17.0/1000 in 2014, but it decreased to 15.6/1000 in 2016.

In 2010, ASD prevalence was slightly lower in 4-years than in 8-years children (13.4 vs. 14.7/1000; this gap seemed to be bridged in 2014 (17.0 vs. 16.8/1000 in 4-years vs. 8-years children), suggesting an improvement in early diagnosis of ASD, but it appeared again in 2016 (15.6 vs. 18.5/1000 in 4-years vs. 8-years children).

The increase of prevalence from 2008 to 2016 corresponds to a variation in the distribution of ASD-diagnosed children with respect to the intellectual disability (ID; for details see Table 7. The proportion of ASD subjects with moderate ID remains constant across calendar years (24–25%), while the proportion of children with severe ID decreases (from 38 to 31–33%) and that of children without ID increases (from 38 to 42–46%). The prevalence increase with respect to 2008 in children grouped by IQ level suggests the hypothesis that a large part of the increase in the overall prevalence depends on the increase in children without ID, likely due to a greater ability to recognize children with milder forms of ASD (including high-functioning autism and Asperger’s syndrome).

In 2018, Xu et al. reported an estimate of ASD prevalence of 24.7/1000 in children and adolescent aged 3–17 years in the 2014–2016 period, based on data from the National Health Interview Survey (NHIS), an annual health survey in the USA [56]. Similar prevalence was obtained by Kogan et al. [57] using data from the National Survey of Children’s Health (NSCH) to estimate the national prevalence of parent-reported ASD diagnoses in US children from 3 to 17 years of age in 2016. The estimated value was 25.0/1000 in the overall group of children, and 26.1/1000 in children aged 6-11 years. Both NHIS and NSCH are nationwide surveys (the latter based on a larger sample than the former), and thus potentially representative of the whole country; however, data come from parents, who are asked to report if their targeted child was ever told to have ASD by a doctor or health professional. This introduces possible report biases with not quantifiable effects on the prevalence estimate: for this reason, data are not comparable with those coming from the ADDM Network surveys.

In Mexico, a survey on children aged 8 years was performed in 2011-2012 in the city of Leon in Guanajuato [42]. Subjects enrolled in the study were students of regular (GSS) or special education (SEMR) schools. Children from GSS underwent a screening phase based on the Social Responsiveness Scale filled in by parents or teachers, and when the score passed the threshold, they were invited to undergo a diagnostic assessment. Based on these data, ASD prevalence was estimated to be 8.70/1000, lower than the prevalence estimated in USA in the same calendar year and quite similar to the USA estimate in 2006.

No prevalence estimates were found for States of Central or South America.

## 3. Factors Potentially Affecting Prevalence

As reported above, Table 2, Table 3 and Table 4 summarize the studies published worldwide after the review by [15], concerning prevalence estimates in children and adolescents from 1 to 17 years of age (*n* = 42 studies). Prevalence estimates still vary across and within geographical areas, countries, year of study and source of data used in the study to estimate the prevalence. To detect the contribution of these factors on the variability observed in the prevalence estimates, we performed simple and multiple regression analyses. Specifically, studies were divided into two subgroups according to the age range of subjects (*Agerange*): age group 1 = age range including 7–8 years and/or lower limit of the age range above 5 years (*n* = 36); age group 2 = upper limit of the age range up to 5 years (*n* = 6). Prevalence was the dependent variable, while geographical area (*Area*: America, Asia, Australia, Europe, and Middle East), source of data (*Source*: administrative data coming from one or multisource databases, ad hoc study, and report), *Agerange* and year of study performance (*Year*) were the independent variables. As for *Year*, some studies report estimates referring to one specific calendar year (*n* = 28), in others estimates refer to periods of two (*n* = 7) or four or more years (*n* = 3), and others do not specify the calendar year of study performance (*n* = 4). When more than one year was indicated, we imputed the most recent year of the interval, and when no year was reported we imputed the year before that of study publication. Europe was used as reference level for *Area*, administrative data for *Source*, and age range including 7 and/or 8 years for *Agerange*. Since more studies could be performed in a single country within a geographical area, *Country* (e.g., Italy, France, within Europe area; Oman, Iran within Middle East area, etc.) was considered as clustering factor. The effect of *Year of study* on the prevalence estimate was evaluated in the simple regression. Since, as reported above, *Year* could not be determined with sufficient precision in the 17% of studies, and the effect of *Year* on prevalence estimates in the simple regression analysis was not significant, we did not include this variable in the multiple regression analysis. Variance inflation factors (VIF) were computed for any variable included in the multiple regression model; all VIF values were lower than 5, thus supporting the absence of multicollinearity. The results of regression analyses are presented in Table 8.

The regression analyses present obvious limitations, due to the low number of studies (*n* = 42), especially in relation to the large number of combinations of area, source of data, and age of children levels (5 × 3 × 2 = 30 different combinations), making it difficult to disentangle the effects of the different factors. However, we can draw some indication on the potential explanatory factors affecting prevalence estimates.

From the simple regression analyses a significant difference among *Areas* was observed, with Europe showing significantly lower prevalence with respect to Australia (*p* < 0.001). Prevalence estimated based on parents’ or teachers’ reports was significantly higher than prevalence estimated by administrative data (*p* = 0.044), while neither *Agerange* nor *Year* of study performance significantly affected prevalence estimates.

Multiple regression model (see Table 8) confirms that studies that use parents’ and teachers’ report predict higher prevalence in respect with administrative data (*p* < 0.001). On the contrary, since the estimates from Asia were mainly based on ad hoc studies, and those from Australia were both based on reports, when accounting for the source of data prevalence estimates from both areas turned to be significantly lower than those from Europe (*p* = 0.029 and *p* < 0.001 for Asia and Australia, respectively). Finally, estimates in younger children turned out to be significantly higher than estimates in older subjects (*p* = 0.007). Overall, the factors included in the multiple regression model explained about 54% of the variance in prevalence estimates (*R^2^* = 0.5314), notwithstanding that the number of independent variables in the multiple regression model (*n* = 7) was high with respect to the number of studies included (*n* = 42). This suggests the need to investigate other variables, likely related to exposure to different risk factors for autism, in order to explain the observed variability.

## 4. Discussion

The analysis of the literature on ASD prevalence studies published since 2014 confirms a high variability of prevalence estimates worldwide. This variability is still accompanied by methodological differences among the performed studies that concern how cases are detected, which population is involved in, and, to a lesser measure, how cases are defined.

Interestingly, the longitudinal analysis of data across years within the same geographical area confirms the increase of prevalence estimates that has repeatedly drawn scientists’ attention in the last twenty years [1]. Studies from Australia [38], Canada [39], Oman [24,25], and USA (see Table 5 and Table 6) and some European countries (Sweden, [16]; Italy, [20]) show a substantial increase of ASD prevalence estimates over the years especially at the turn of the 2010. However, the consistency of the increase over countries is masked by the high variability of the prevalence estimates (see Table 2, Table 3 and Table 4) over the continents, with a range from 0.8/1000 in the North, Sirajganj district of Bangladesh to 93/1000 in Japan.

As previously reported, one of the putative methodological issues contributing to the high variability of ASD prevalence is the source of data from which ASD cases have been detected. From the present analysis, it emerges that the main sources of ASD cases are administrative data (mono or multisource), ad hoc studies, and surveys based on questionnaires. The simple and multiple regression analyses show that the source of data indeed affects the estimate of ASD prevalence. In particular, when ASD cases are detected by teachers’ or parents report, prevalence estimated seem to be significantly overestimated. Otherwise, one or more phased population studies appear to produce higher prevalence estimate then studies based on administrative data, but the difference apparently is not significant.

As previously evidenced [58], it is possible that the methodological and qualitative advantages and disadvantages of the use of different sources of data, make it difficult to choose a specific surveillance policy about the count of ASD cases. Population-based designs are considered a high research standard because they are representative of all children in defined populations who meet selected ASD criteria and are evaluated in “natural” community settings, rather than of selected samples attending a particular setting (clinic or educational) or registered in specific research projects. However, the source of identification (teacher; parent; professionals), the lack of blindness of the assessor, the multi-phasing of the study (in relation to the sensitivity of the screening tools and the specificity of the confirmation diagnostic tools) as well as the sample setting (e.g., mainstream vs. special school [29]), and the case definition [59], all represent potential biases that may affect the prevalence estimate obtained by population studies. Otherwise, as above stated, estimates based on administrative classifications have other limitations, due to either difference between states in administrative policies and regulations for the access to the system of recording [60], and/or to socioeconomic disparities or different services availability over the countries [8,9]. Finally, as noted by some scholars, in the survey-based prevalence studies, the formulation of the questionnaire or interview to be administered to parents or teachers can influence the understanding of the questions asked [58] also taking into account educational and/or cultural factors. Furthermore, recall-bias is an intrinsic limitation of this kind of study.

Results of multiple regression also highlight differences in prevalence due to the geographical area where the study is performed, with Europe showing significantly lower prevalence than Australia and Asia. As argued above, this result appears to be at least partially due to the source of data used in the study, but it can indeed be due to several determinants such as socio-cultural [61,62] and socio-economic factors [63], including organisational factors [37,64].

As seen above, factors such as case definition and case-finding procedures, and geographical area, however, scholars reported that they appear to account only for about a 50% of the variability, thus suggesting that additional factors linked to the aetiology of ASD should be considered in explaining variability of ASD prevalence across areas and over time [65,66,67]. Current literature suggests that several environmental factors could affect brain development and differentiation over perinatal period resulting in neurodevelopmental disorders emerging at different time life. These studies overall focus on dynamic interactions between biological and non-biological risk factors [68]. As for ASD, CHARGE (Childhood Autism Risks from Genetics and the Environment) study is an excellent example of epidemiological study contributing to the understanding of which factors can increase the risk of ASD. Three groups of children have been enrolled in the CHARGE study: children with autism, children with developmental delay but without autism and children from the general population. All of them are evaluated for a broad array of exposures and susceptibilities [69]. Among evidences obtained through the analysis of data collected by the ASD group of children, folate prenatal intake, maternal fever, pesticides exposure, and air pollution, seem to be associated with an ASD risk increase [70]. However, CHARGE study adopts a retrospective case-control approach that ranks in the lower level of the pyramid evidence. Other evidence came from cohort studies that highlighted others possible risk factor such as parental age at birth [71]. Furthermore, some maternal factors (i.e., maternal age, pregnancy and delivery condition, drug intake, maternal autoimmunity, inflammation and chronic stress) are of increasing concern and suggest the need of further studies [72].

In conclusion, multiple and complementary systems are needed to better estimate ASD prevalence and to understand its observed changes. It is necessary to establish either surveillance systems in order to monitor the change of prevalence with time, or guidelines for the performance of ad hoc studies to compare the prevalence across geographical areas. Although the reliability of the prevalence estimates coming from the ADDM Network has been questioned [73], until now this is the only surveillance systems that tracks ASD prevalence over the years and across states, allowing to study factors that possibly give reasons for the observed prevalence increase. Finally, methodological differences across studies could not fully account for the large variation among the prevalence estimates. This suggests the need tostudy other factors, pertaining to the capability to recognize and diagnose ASD and/or to the exposure to genetic and environmental risk factors for ASD, in order to explain the prevalence variation.

## Figures and Tables

**Table 1:** Summary of PDD prevalence estimates of the studies included in the reviews by [7,15] *.

	From [7]				From [15]				
	*N* Studies	Publication Year	Prevalence (/1000)		*N* Studies	Publication Year	Prevalence (/1000)		Papers Examined by [15] Already Included in [7] (%)
		Range	Median	Range		Range	Median	Range	
Europe	14	2000–11	6.16	3.0 to 11.6	21	2000–12	6.19	3.0 to 12.3	66.7
Middle East	3	2007–12	0.63	0.14 to 2.9	4	2007–12	1.76	0.14 to 24.0	75.0
Asia	4	2008–11	14.41	1.6 to 18.9	6	2008–12	6.50	1.4 to 26.4	66.7
Australia & New Zealand	1	2004	3.92	--	2	2004–09	3.15	2.4 to 3.9	50.0
North America	10	2001–10	6.65	3.4 to 11.0	24	2001–14	7.17	0.21 to 17.4	33.3
Central & South America	3	2008–10	2.72	1.3 to 5.3	4	2008–11	3.99	1.7 to 5.3	75.0
Africa	0	--	--	--	0	--	--	--	

* [7] presented the prevalence estimates for the diagnostic categories Autistic Disorder (AD) and Pervasive Developmental Disorder (PDD), which is the diagnostic category that evolved to ASD passing from DSM-IV to DSM5; [15] used AD, and PDD or ASD. We report only the prevalence estimates for PDD (or ASD).

**Table 2:** Summary of prevalence studies published since 2014 in Europe and Middle-East.

Country	Extent of Coverage	Area/Region	Year of Prevalence Estimate	Age (Years)	ASD Detection Based On	Prevalence/1000	95% CI	Reference
***Europe***								
Sweden	Regional	Stockholm County	2011	6–12	multisource	17.4	16.8 to 18.0	[16]
Poland	Regional	West Pomerania WP	2010–2014	4–7	administrative data	5.4	4.8 to 5.9 *	[16]
		Pomerania P	2010–2014	4–7	administrative data	5.2	4.8 to 5.7 *	[17]
		Overall WP+P	2010–2014	4–7	administrative data	5.3 *	5.0 to 5.6 *	[17]
Germany	National		2012	6–11	administrative data	6.0	na	[18]
Denmark	National		2015	8	administrative data	12.6	11.7 to 13.5	[19]
Finland	National		2015	8	administrative data	7.7	7.0 to 8.4	[19]
France	Regional	South-West	2015	8	administrative data	7.3	6.0 to 8.7	[19]
		South-East	2015	8	administrative data	4.8	4.0 to 5.6	[19]
Iceland	National		2015	8	administrative data	31.3	26.4 to 36.8	[19]
Italy	Regional	Tuscany	2015	7–9	ad hoc: TN, SCQ, ADOS, clinical assessment	11.5	8.3 to 14.6	[20]
Italy	Regional	Piemonte	2016	6–10	administrative data	4.2	na	Admin regional reports
Italy	Regional	Emilia-Romagna	2016	6–10	administrative data	4.3	na	Admin regional reports
Italy	Regional	Abruzzo	2018	6–8	administrative data	8.0	6.0 to 10.0	[21]
*Spain*	*Regional*	*Tarragona*	*na (<2018)*	*3–5*	*ad hoc: 1. CAST, EDUTEA, 2. ADI-R, ADOS*	*15.5*	*8.96 to 22.0*	[22]
Spain	Regional	Catalonia	2017	6–10	administrative data	11.8	11.4 to 12.1	[23]
***Middle East***								
Iran	National		na (≤2016)	6–9	ad hoc: K-SADS	1.1	0.4 to 1.7	[24]
Oman	National		2011–2018	5–9	administrative data from diagnostic centers	2.0	1.9 to 2.2	[25]
Qatar	National		2015–2018	6–11	ad hoc: 1. SCQ, 2. QSS-PTI, clinical assessment, QCC-AF	11.4	8.9 to 14.6	[26]
*Lebanon*	*Regional*	*Beirut and Mount Lebanon*	*2014*	*1.3–4*	*ad hoc: M-CHAT*	*15.3*	*7.7 to 22.9*	[27]

* Prevalence estimates and/or 95%CIs calculated from information reported in the original papers. Prevalence estimates in children aged less than 5 years are reported in italic.

**Table 3:** Summary of prevalence studies published since 2014 in Asia, Australia &New Zealand.

Country	Extent of Coverage	Area/Region	Year of Prevalence Estimate	Age (Years)	ASD Detection Based on	Prevalence/1000	95% CI	Reference
***Asia***								
*China*	*Regional*	*Tiajin*	*2009–2010*	*1.5–3*	*ad hoc: 1. M-CHAT, 2. DSM IV*	*2.8*	*1.6 to 3.9*	[28]
China	Regional	Beijing	na (<2015)	6–11	ad hoc: 1. CAST, 2. ADOS and ADI-R; mainstream schools	11.9	3.9 to 19.9 *	[29]
China	Regional	Jilin	2013	6–10	ad hoc: 1. CAST, 2. clinical assessment; mainstream /special schools /other settings	10.8	8.7 to 13.5	[30]
		Jilin		6–10	ad hoc: 1. CAST, 2. clinical assessment; mainstream schools	1.5	0.5 to 2.4 *	[30]
		Shenzhen	2013	6–10	ad hoc: 1. CAST, 2. clinical assessment; mainstream schools	4.2	2.0 to 8.9	[30]
		Jiamusi	2013	6–10	ad hoc: 1. CAST, 2. clinical assessment; mainstream schools	1.9	1.0 to 3.8	[30]
*China*	*Regional*	*Shenzhen*	*2014*	*3.8–4.8*	*ad hoc: 1. ABC; mainstream kindergartens*	*26.2*	*23.7 to 28.7*	[31]
Japan	Regional		2015	6–9	parents’ report data, SRS	19.0	13.0 to 25.0	[32]
			2015	6–9	teachers’ report data, SRS	93.0	72.0 to 118.0	[32]
India	Regional	South (Kerala)	2011-2012	6–10	ad hoc: screening by questionnaire	5.0	2.5 to 7.6 *	[33]
India	Regional	Kolkata	2013	3–8	ad hoc: 1. SCDC, 2. SCQ, 3. ADO Smainstream and special schools	2.3	0.7 to 4.6	[34]
India	Regional	Northwest (Himachal Pradesh)	na (<2017)	1–10	ad hoc:1. ISAA, 2, clinical assessment	1.5	1.1 to 2.0	[34]
Nepal	Regional	Makwanpur district	2014-2015	9–13	ad hoc: AQ-10 screening tool	3.4	1.6 to 5.2	[35]
*Bangladesh*	*Regional*	*North, Sirajganj district*	*2016*	*1.5–3*	*ad hoc: 1. M-CHAT, 2. DSM IV*	*0.8*	0.0 to 1.5 *	[36]
*Vietnam*	*Regional*	*North*	*2017*	*1.5–2.5*	*ad hoc: 1. M-CHAT, 2. DSM IV*	10.8 *	9.3 to 12.4 *	[37]
***Australia & New Zealand***								
Australia	National		2005–2006	6–7	parents’ report data on diagnoses	14.1 *	10.5 to 17.6 *	[38]
			2010–2011	6–7	parents’ report data on diagnoses	25.2	20.0 to 30.0	[38]

* Prevalence estimates and/or 95%CIs calculated from information reported in the original papers. Prevalence estimates in children aged less than 5 years are reported in italic.

**Table 4:** Summary of prevalence studies published since 2014 in North America.

Country	Extent of Coverage	Area/Region	Year of Prevalence Estimate	Age (Years)	ASD Detection Based On	Prevalence /1000	95% CI	Reference
***North***								
Canada	Regional	Newfoundland and Labrador	2008	6–9	multisource	10.8	9.4 to 12.3	[39]
		Prince Edward Island	2010	6–9	multisource	10.0	7.6 to 12.9	[39]
		Southeastern Ontario	2010	6–9	multisource	16.2	14.5 to 18.1	[39]
Canada	Regional	Quebec	2014–2015	1–17	multisource	12.2	na	[40]
USA	Regional	11 States	2014	8	multisource, evaluated by DSM-IV	16.8	16.4 to 17.3	[12]
	Regional	11 States	2016	8	multisource, evaluated by DSM-V	18.5	18.0 to 19.1	[41]
Mexico	Regional	Leon city, Guanajuato	2011–2012	8	ad hoc: 1. parents’ and teachers’ SRS, 2. ADOS and ADI-R	8.7	6.2 to 11.0	[42]

**Table 5:** CDC-ADDM Network ASD prevalence estimates per 1000 children aged 8 years, in the overall group and in sex and ethnicity subgroups, from 2000 to 2016 in USA [12,41,50,51,52,53,54,55].

Study Year	2000	2002	2006	2008	2010	2012	2014	2016
States (nr.)	6	14	11	14	11	11	11	11
Population	187,761	407,578	308,038	337,093	363,749	346,978	325,483	275,419
Prevalence								
Overall	6.7 (*1:150*)	6.6 (1:150)	9.0 (1:110)	11.3 (1:88)	14.7 (1:68)	14.6 (1:68)	16.8 (1:59)	18.5 (1:54)
Range	4.5 WV 9.9 NJ	3.3 AL 10.6 NJ	4.2 FL 12.1 AZ, MO	4.8 AL 21.2 UT	5.7 AL 21.9 NJ	8.2 MD 24.6 NJ	13.1 AR 29.3 NJ	13.1 CO 31.4 NJ
IQ								
% IQ ≤70	(40%–62%)	45% (33%–59%)	41% (29%–51%)	38% (13%–54%)	31% (18%–37%)	32% (20%–50%)	31% (27%–39%)	33% (25%–42%)
Sex								
Males	*10.3*	*10.2*	14.5	18.4	23.7 (1:42)	23.6 (1:42)	26.6 (1:38)	29.7 (1:34)
Females	*2.9*	*2.4*	3.2	4.0	5.3 (1:189)	5.3 (1:189)	6.6 (1:152)	6.9 (1:145)
M:F	*3.6:1*	*4.2:1*	4.5:1	4.6:1	4.5:1	4.5:1	4.0:1	4.3:1
Ethnicity								
White, non-Hispanic	4.5–11.3	*7.0*	9.9	12.0	15.8	15.5	17.2	18.5
Black, non-Hispanic	5.3–10.6	*5.5*	7.2	10.2	12.3	13.2	16.0	18.3
Hispanic	*na*	*3.7*	5.9	7.9	10.8	10.1	14.0	15.4
Asian/Pacific Islander	*na*	*na*	*na*	*na*	12.3	11.3	13.5	17.9

AL Alabama, AR Arkansas, AZ Arizona, CO Colorado, FL Florida, MD Maryland, MO Missouri, NJ New Jersey, UT Utah, WV West Virginia. Italics indicates data computed from information reported in original papers.

**Table 6:** CDC-ADDM Network ASD prevalence estimates per 1000 children aged 4 years, in the overall group and in sex and ethnicity subgroups, from 2000 to 2016 in USA [1,42].

Study Year	2010	2012	2014	2016
States (nr.)	5	5	6	6
Population	58,467	59,456	70,887	72,277
Prevalence				
Overall	13.4 (1:73)	15.3 (1:65)	17.0 (1:59)	15.6 (1:64)
Range	8.5 MO–19.7 NJ	8.1 MO–22.1 NJ	9.6 MO–28.4 NJ	8.8 MO–25.3 NJ
IQ				
% IQ ≤70	47.0%	43.6%	46.1%	52.6%
Sex				
Males	12.2 MO–31.7 NJ	12.9 MO–33.6 NJ	14.2 MO–44.0 NJ	13.4 MO–38.7 NJ
Females	4.6 MO–7.3 AZ	3.2 MO–9.9 NJ	4.3 CO–12.1 NJ	3.9 MO, NC–11.0 NJ
M:F	2.6–4.4:1	3.4–4.7:1	3.0–5.2:1	3.1–4.9:1

AL Alabama, AR Arkansas, AZ Arizona, FL Florida, MD Maryland, MO Missouri, NJ New Jersey, UT Utah, WV West Virginia.

**Table 7:** CDC-ADDM Network ASD prevalence estimates per 1000 children of 8 years of age, in the overall group and in IQ subgroups, from 2008 to 2016 in USA, and percent variation of prevalence with respect to 2008 data (values are computed from original data in [8,12,52,53,55]).

	2008		2010		2012		2014		2016		2010 vs. 2008	2012 vs. 2008	2014 vs. 2008	2016 vs. 2008
	%	Prev	%	Prev	%	Prev	%	Prev	%	Prev	%	%	%	%
Overall	100	11.3	100	14.7	100	14.6	100	16.8	100	18.5	+29.5	+28.8	+48.4	+63.7
IQ > 85	38	4.31	46	6.75	43.9	6.41	44	7.40	42.1	7.85	+56.8	+48.8	+71.8	+82.2
IQ 71–85	24	2.72	23	3.38	24.5	3.57	25	4.20	24.1	4.51	+24.1	+31.4	+54.6	+65.7
IQ ≤ 70	38	4.31	31	4.55	31.6	4.61	31	5.21	33.4	6.19	+5.6	+7.1	+21.0	+43.8

**Table 8:** Results of simple and multiple regression analyses performed on the selection of studies (*n* = 42) listed in Table 2, Table 3 and Table 4.

Model		Simple Regression						Multiple Regression					
	*N* studies	Coefficient	95	%	CI	*p*	*R* ^2^	Coefficient	95	%	CI	*p*	*R* ^2^
**Area**													
*vs* Europe	14						0.0302						0.5314
America	7	2.76	−3.01	to	8.54	0321		3.21	−2.63	to	9.05	0.254	
Asia	15	2.46	−12.82	to	17.74	0.739		**−5.58**	**−10.87**	to	**−0.29**	**0.029**	
Australia	2	**9.45**	**4.78**	to	**14.12**	**<0.001**		**−41.58**	**−46.87**	to	**−36.29**	**<0.001**	
Middle East	4	−3.10	−11.32	to	5.13	0.435		−4.18	−10.29	to	1.93	0.156	
**Source**													
*vs* Admin + Multisource	19						0.3472						
Ad hoc assessment	19	−3.19	−8.02	to	1.64	0.171		−0.06	−5.60	to	5.47	0.981	
Report	4	**27.09**	**0.14**	**to**	**54.05**	**0.044**		**51.47**	**45.25**	to	**57.68**	**<0.001**	
**Agerange**													
*vs* Age group 1 ^1^	36						0.0000						
Age group 2 ^2^	6	−0.18	−8.48	to	8.12	0.965		**6.26**	**1.47**	to	**11.05**	**0.007**	

^1^ Age group 1 = age range including 7–8 years and/or age range all above 5 years; ^2^ Age group 2 = age no more than 5 years. Significant effects are highlighted in bold.
